# A bizarre electrocardiogram with a fruitful recovery

**DOI:** 10.1007/s12471-024-01875-7

**Published:** 2024-05-24

**Authors:** Anna van Veelen, Joëlle Elias, Pieter G. Postema, Mariëlle C. van de Veerdonk

**Affiliations:** Department of Cardiology, Amsterdam University Medical Centres, Amsterdam Cardiovascular Sciences Research Institute, University of Amsterdam, Amsterdam, The Netherlands

A 44-year-old female presented to the emergency room after falling down the stairs at home for an unknown reason. The patient was found by her partner 6 h after the fall, whereupon he called for an ambulance. The cardiologist was consulted because of her abnormal electrocardiogram (Figs. 1 and 2). The patient had a history of depression, for which she did not take medication. There was no cardiac history, and no prior electrocardiograms were available for comparison. She complained of nausea and vomiting. During trauma assessment using the ABCDE approach, the patient’s airways and breathing were stable without support. Circulation was hampered by bradycardia of around 30 bpm and hypotension with a systemic blood pressure of 70/50 mm Hg, for which norepinephrine was uptitrated. Disability assessment revealed impaired consciousness, with a Glasgow Coma Score of 13 (E3M6V4) and time disorientation. Exposure showed facial lacerations. The paramedics reported not seeing pills or drugs at her home that may have caused intoxication.

1. What is your differential diagnosis based on the electrocardiographic findings?
2. What would be your next steps or what treatment would you initiate?

**Fig. 1:**
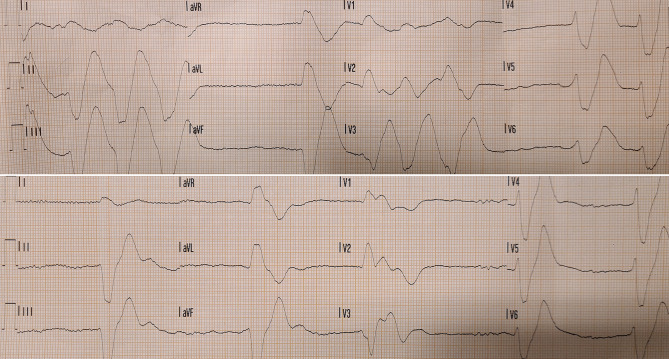
Ambulance electrocardiograms (25 mm/s, 10 mm/mV)

**Fig. 2:**
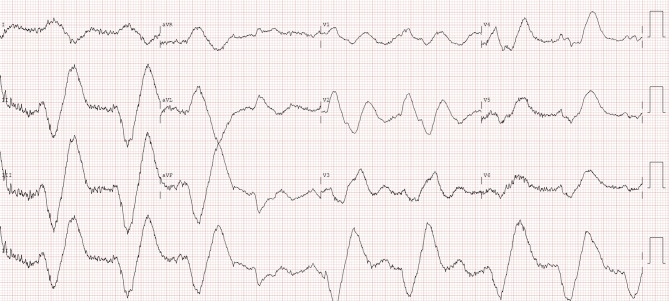
Electrocardiogram obtained in trauma room (25 mm/s, 10 mm/mV, 0.05 Hz high-pass filter for baseline noise removal, 150 Hz low-pass filter for high-frequency noise suppression)

## Answer

You will find the answer elsewhere in this issue.

